# Studies of a Large Odd‐Numbered Odd‐Electron Metal Ring: Inelastic Neutron Scattering and Muon Spin Relaxation Spectroscopy of Cr_8_Mn

**DOI:** 10.1002/chem.201503431

**Published:** 2016-01-08

**Authors:** Michael L. Baker, Tom Lancaster, Alessandro Chiesa, Giuseppe Amoretti, Peter J. Baker, Claire Barker, Stephen J. Blundell, Stefano Carretta, David Collison, Hans U. Güdel, Tatiana Guidi, Eric J. L. McInnes, Johannes S. Möller, Hannu Mutka, Jacques Ollivier, Francis L. Pratt, Paolo Santini, Floriana Tuna, Philip L. W. Tregenna‐Piggott, Iñigo J. Vitorica‐Yrezabal, Grigore A. Timco, Richard E. P. Winpenny

**Affiliations:** ^1^ School of Chemistry and Photon Science InstituteThe University of ManchesterOxford RoadManchesterM13 9PLUK; ^2^ Institut Laue-Langevin, BP 1566 rue Jules Horowitz38042Grenoble Cedex 9France; ^3^ Department of PhysicsDurham UniversitySouth RoadDurhamDH1 3LEUK; ^4^ Dipartimento di Fisica e Scienze della TerraUniversità di Parma43124ParmaItaly; ^5^ ISIS FacilityRutherford Appleton LaboratoryDidcotOX11 0QXUK; ^6^ Clarendon LaboratoryDepartment of PhysicsUniversity of OxfordOX1 3PUUK; ^7^ Department of Chemistry and BiochemistryUniversity of Bern3000BernSwitzerland

**Keywords:** inelastic neutron scattering, molecular magnetism, muon spin relaxation, spin chirality, spin frustration

## Abstract

The spin dynamics of Cr_8_Mn, a nine‐membered antiferromagnetic (AF) molecular nanomagnet, are investigated. Cr_8_Mn is a rare example of a large odd‐membered AF ring, and has an odd‐number of 3d‐electrons present. Odd‐membered AF rings are unusual and of interest due to the presence of competing exchange interactions that result in frustrated‐spin ground states. The chemical synthesis and structures of two Cr_8_Mn variants that differ only in their crystal packing are reported. Evidence of spin frustration is investigated by inelastic neutron scattering (INS) and muon spin relaxation spectroscopy (μSR). From INS studies we accurately determine an appropriate microscopic spin Hamiltonian and we show that μSR is sensitive to the ground‐spin‐state crossing from *S*=1/2 to *S*=3/2 in Cr_8_Mn. The estimated width of the muon asymmetry resonance is consistent with the presence of an avoided crossing. The investigation of the internal spin structure of the ground state, through the analysis of spin‐pair correlations and scalar‐spin chirality, shows a non‐collinear spin structure that fluctuates between non‐planar states of opposite chiralities.

## Introduction

Paramagnetic cage complexes, sometimes called molecular nanomagnets (MNMs), have been extensively studied^1^ due to their interesting physics, and to the possibility of exploiting their magnetic behaviour in technological applications.^2^ Cyclic compounds have been investigated since the early 1990s, beginning with studies of an Fe_10_ ring, which showed steps in the magnetisation on increasing the applied magnetic field.^3^ Later work has included studies of the Néel vector^4^ tunneling, observation of quantum fluctuations of total spin at avoided crossings,^5^ the first measurements of coherence times in molecular magnets,^6^ and the demonstration that these coherence times can be modified by chemistry.^7^ It has also been possible to investigate spin dynamics at the atomic scale in such cyclic metal cages using single‐crystal inelastic neutron scattering (INS) studies.^8^

The studies listed above all involve even‐membered metal rings. When the magnetic exchange is antiferromagnetic (AF), a simple picture can be used to describe the magnetism, with spins on individual metal centres aligned alternatively up and down. There are far fewer reports of large odd‐numbered metal rings, in which such a simplistic picture must fail, and the physical studies reported are even more limited. Examples include the magnetisation studies of a (VO)_7_ ring,^9^ and a cyclic Fe_9_ phosphonate.^10^ Our own work has included investigations of a Cr_8_Ni ring, which was visualised as a magnetic Möbius strip,^11^ and later as a rare example of a valence‐bond solid.^12^ Cr_8_Ni has an odd‐number of metal ions in the ring, but has an even number of unpaired electrons giving rise to an *S*=0 singlet ground state. In some definitions of spin frustration, for example, that given by Kahn,^13^ Cr_8_Ni cannot be frustrated due to its lack of ground‐state degeneracy. In a more recent work, Schnack has attempted to define frustration by considering whether the ground‐state spin structure is bipartite (i.e., alternately spin up and down) or not.^14^ With this definition Cr_8_Ni is frustrated. We have also proposed an alternative approach in work on slightly distorted Cr_9_ rings,^15^ in which we define frustration as Type 1—which is the strongest case, in which the ground state is degenerate (Kahn′s definition); Type 2—which is essentially Schnack′s definition, in which the spin ground state cannot be described by coupling spin sites classically, but in which degeneracy is not required; and Type 3, in which the ground state can be described by coupling spin sites classically, but in which competing antiferromagnetic interactions are necessary for the description of the system spin dynamics. The last case would then include a number of claims of “frustration” from the chemical literature that would not be allowed by either the Kahn or Schnack definitions.

Our new categorisation was based on studies of two chemically similar Cr_9_ rings by INS and magnetometry.^15^ One had two low‐lying *S*=1/2 states and the other had an *S*=3/2 ground state, and hence they belong to spin frustration classifications Type 2 and Type 3, respectively (we use the convention that capital *S* refers to the total spin state of a molecule, while lower‐case *s* refers to the spin of individual ions). Here we discuss the case of a heterometallic nine metal ring, [H_2_N^*i*^(C_3_H_7_)_2_][Cr_8_MnF_9_(O_2_C*t*Bu)_18_] (**1**; Cr_8_Mn), which also has both an odd number of metal ions and an odd number of electrons. We describe the synthesis and structure of the compound, and derive the microscopic spin Hamiltonian and the energy spectrum of this molecule by means of magnetisation and INS studies. We then use muon spin relaxation (μSR) spectroscopy to probe the nature of the spin ground state as a function of applied magnetic field and to investigate the crossing of energy levels with different total‐spin quantum numbers. μSR spectroscopy has previously been shown to be an ideal tool for studying magnetic phase transitions and spin fluctuations.^16^ Finally, we exploit the experimentally parameterised Hamiltonian model to investigate the ground state of Cr_8_Mn and identify that the internal spin structure fluctuates between opposite chiralities.

## Results and Discussion

### Synthesis and structure

The compound [H_2_N^*i*^(C_3_H_7_)_2_][Cr_8_MnF_9_(O_2_C*t*Bu)_18_] (**1**) can be obtained from the reaction of hydrated chromium(III) fluoride with pivalic acid in the presence of diisopropylamine, followed by addition of manganese carbonate [Eq. (1)].

(1) $$\mathrm{CrF}{}_{3}\cdot \;4\;H{}_{2}O+t\mathrm{BuCO}{}_{2}H+\mathrm{MnCO}{}_{3}+\mathrm{HN}{}^{i}\left(C{}_{3}H{}_{7}\right){}_{2}\to \left[H{}_{2}N{}^{i}\left(C{}_{3}H{}_{7}\right){}_{2}\right]\left[\mathrm{Cr}{}_{8}\mathrm{MnF}{}_{9}\left(O{}_{2}Ct\mathrm{Bu}\right){}_{18}\right]$$

The reaction proceeds to give **1** in a relatively good yield (42 %, based on Cr), but crystallisation presents an interesting problem. The green compound can be crystallised readily from a pentane/toluene mixture, giving large hexagonal crystals of compound **1 a**. These crystals do not diffract X‐rays well, and the diffraction pattern is only sufficiently resolved to establish the formation of nine‐membered metal rings, with these rings all co‐planar and the plane of the rings perpendicular to the unique axes of the hexagonal crystals. A very similar problem was found for the equivalent Cr_8_Ni ring.11a

By re‐crystallisation of **1 a** using a mixture of EtOAc/MeCN compound **1** crystallises in *P*2_1_/*n* and a structure of **1 b** was obtained at 30 K; refinement gave a structure with an *R* factor of 8 %. The structure was initially refined with all metal sites as Cr; however, the converged structure shows distinct metric differences for one position that was then assigned as the Mn site, and the structure refined to convergence again.

**Figure 1:**
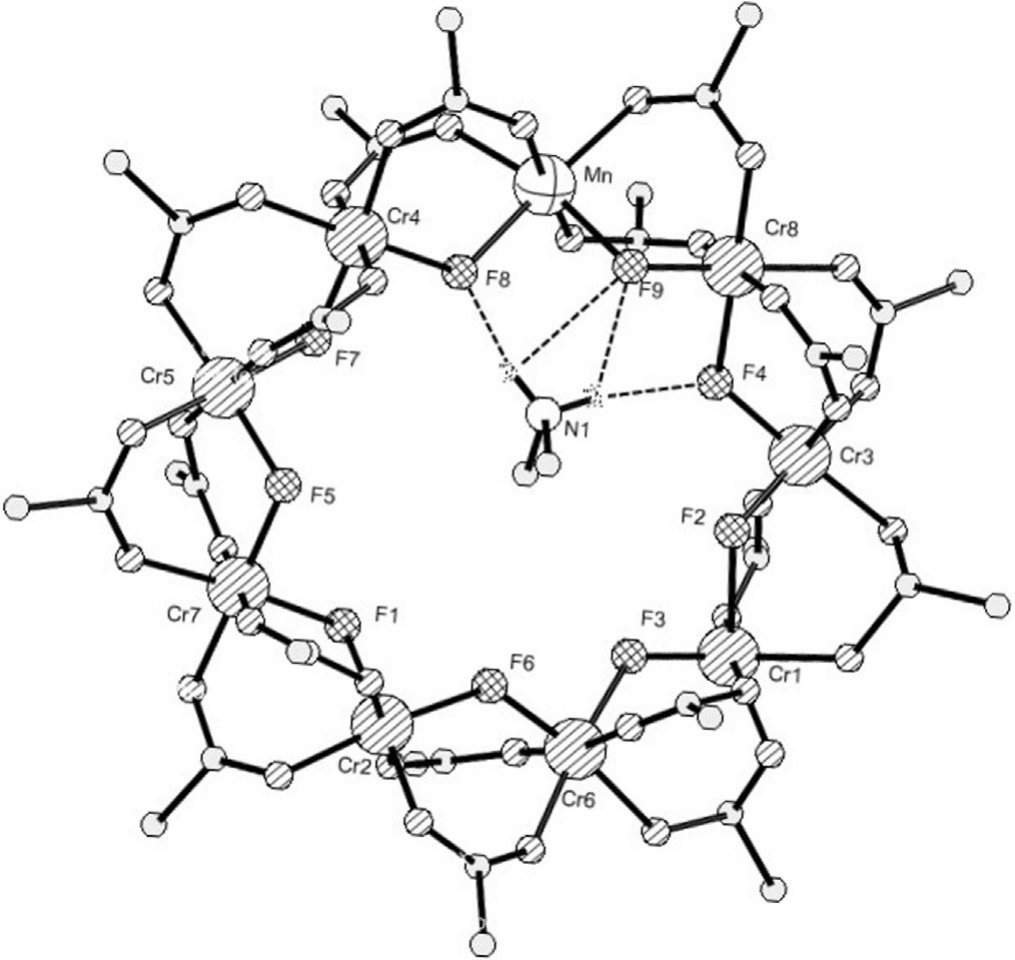
The structure of **1 b** in the crystal. Methyl groups excluded for clarity. Hydrogen atoms excluded for clarity except those on the ammonium cation. Oxygen atoms shaded, carbon atoms open circles.

The structure of **1 b** contains metal sites arranged in a distorted nonagon (or enneagon) (Figure 1). Each metal site is bound to two fluoride and four oxygen donors from carboxylates. For the Cr sites the average Cr−F distances are 1.923±0.060 Å and the Cr−O distances are 1.961±0.064 Å. For the unique Mn site the Mn−F distances are 2.110(3) and 2.180(3) Å, while the Mn−O distances average 2.137±0.026 Å. These differences in bond lengths are clear evidence for Cr^III^ and Mn^II^ sites in the structure, which differs from Cr_7_M rings in which the divalent site is disordered over several sites within the ring.^17^ A fluoride and two pivalate groups bridge each edge of the nonagon. The M⋅⋅⋅M contacts along these edges vary, with Cr⋅⋅⋅Cr edges falling in the range 3.34–3.39 Å, while the two Cr⋅⋅⋅Mn edges are 3.461(1) and 3.509(1) Å. The M⋅⋅⋅M⋅⋅⋅M angles within the nonagon vary from 133 to 143°.

Further evidence for the localisation of the Mn site comes from the position of the central ammonium cation; the N1 atom of this cation is displaced from the centre of the ring towards the Mn site, for example, the N−Mn distance is 3.925(6) Å, compared with the N−Cr6 and N−Cr2 distances, which are 5.967(6) and 5.661(6) Å, respectively. There are N−H⋅⋅⋅F hydrogen bonds to both fluorides bound to the Mn site, with N⋅⋅⋅F distances of 2.763(6) and 2.860(6) Å. The hydrogen bond to a fluoride bridging between a divalent metal and a trivalent metal will be stronger than to a fluoride bridging two trivalent metals as the electron density on the fluoride should be greater. The next shortest N⋅⋅⋅F contact is 3.130(7) Å, to F7.

The metal sites within the nonagon are not all in a single plane, with the mean deviation of the M sites from the plane being 0.183 Å. Three sites—Cr3, Cr4 and Cr2—lie very close to the mean plane (deviation <0.1 Å). In each case the two metals between these sites lie with one significantly above the plane and the other significantly below the plane, for example, Cr8 is 0.35 Å above the plane, while Cr5 is 0.31 Å below. In octametallic Cr_7_M rings the metal octagon is planar. In the octagonal rings one pivalate on each edge lies in the plane of the metal ring, with the other either above or below, alternating around the ring. In **1** this arrangement of carboxylates is impossible as there are an odd number of edges.

The packing of the Cr_8_Mn rings is different between monoclinic and the hexagonal form. In compound **1 b** the rings pack with the mean planes of half the rings at 54° to the others; in the hexagonal form, **1 a**, the rings are all co‐planar.

### Magnetic measurements and inelastic neutron scattering

**Figure 2:**
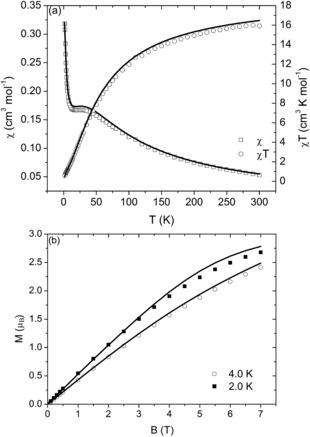
Magnetic measurements on polycrystalline samples of **1 a**. a) *χ*_m_ and *χ*_m_ *T* against *T* recorded in a 0.1 T applied field. b) *M* versus *B* measured at 2 and 4 K.

Magnetic measurements were performed on compounds **1 a** and **1 b** and no difference was observed between the polymorphs; the magnetic properties are molecular and unaffected by the space group. At both 2 and 4 K magnetisation increases with applied magnetic field without reaching saturation within the measured range up to 7 T (Figure 2). At 300 K *χ*_m_ *T* equals 16 cm^3^ K mol^−1^, slightly less than the calculated value of 18.78 cm^3^ K mol^−1^ for eight uncoupled *s*=3/2 and one *s*=5/2 spin with *g*_Cr_=1.96 and *g*_Mn_=2.0. On decreasing temperature the molecular susceptibility (*χ*_m_) steadily increases before flattening off at around 25 K at a value of around 0.17 cm^3^ K mol^−1^, indicating the presence of a significant antiferromagnetic exchange interaction. At lower temperatures, *χ*_m_ increases rapidly confirming the expected non‐zero spin ground state.

**Figure 3:**
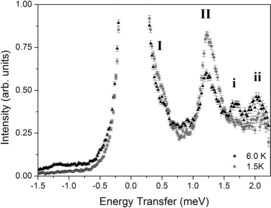
Inelastic neutron scattering intensity of **1 a** as a function of energy transfer measured on the FOCUS spectrometer with a 5.0 Å incident neutron wavelength at 1.5 and 6.0 K. Transition labels are discussed in the text.

The INS energy spectrum of **1 a** was measured with an incident neutron wavelength of 5.0 Å, on the FOCUS spectrometer at 1.5 and 6.0 K (Figure 3). Several magnetic excitations are clearly resolved. Comparison of the spectra at different temperatures indicates a weak excitation in the shoulder of the elastic scattering line, labelled **I**. Subtraction of 1.5–6.0 K data indicates that this excitation comes from the ground state (cold) and is centred at approximately 0.42 meV. An excitation centred at 1.22 meV, labelled **II**, also shows greatest intensity at the base temperature. Two excitations emerge on increasing the sample temperature to 6.0 K, labelled **i** and **ii** at 1.7 and 2.0 meV, respectively. These two excitations originate from a low‐lying excited state and involve transitions to further excited states at higher energies.

**Figure 4:**
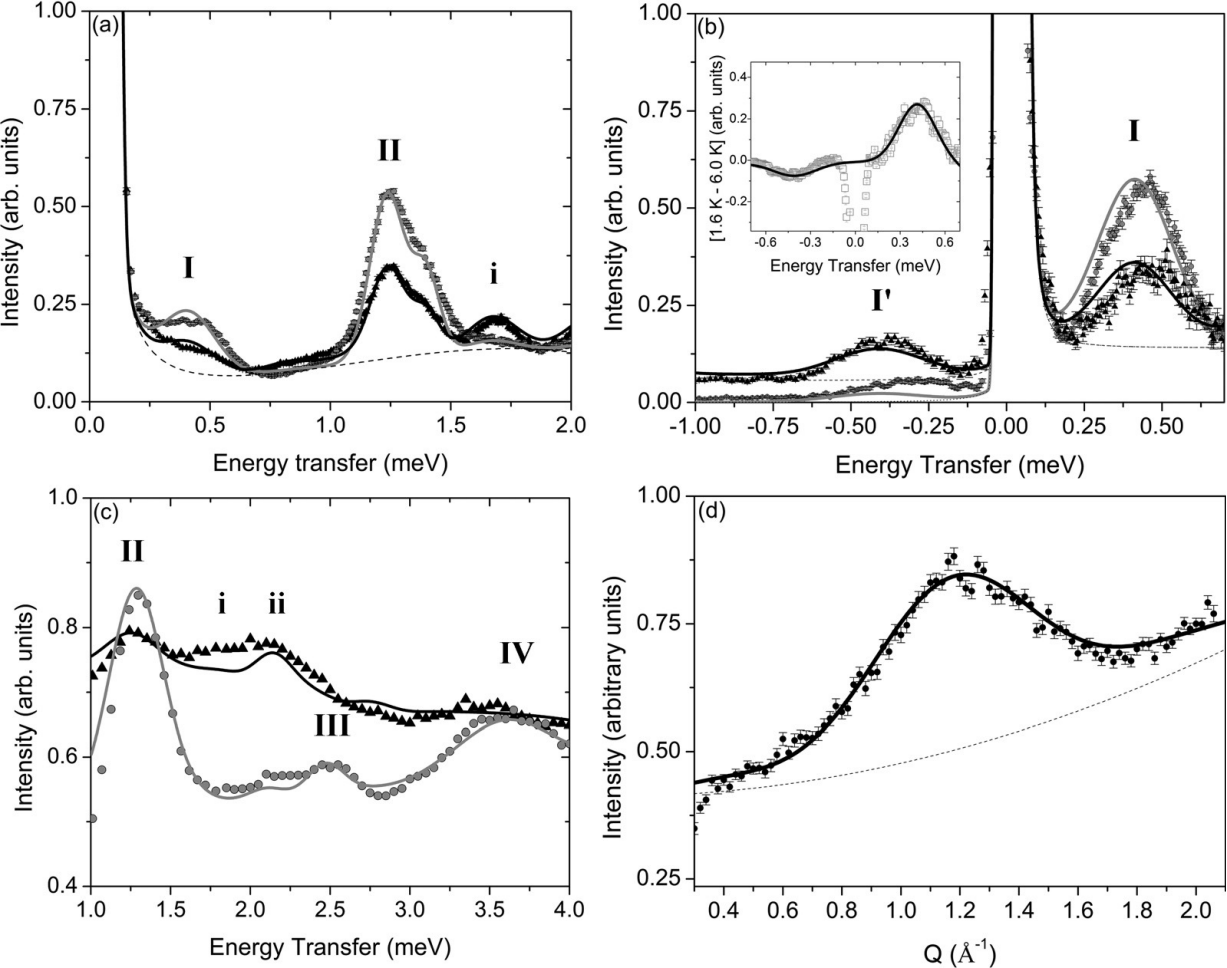
INS spectra of **1 a**. a) 5 Å spectra measured at 1.5 (grey circles) and 6.0 K (black triangles). b) 8.0 Å spectra measured at 1.6 (grey circles) and 6.0 K (black triangles). The insert shows 6.0 K spectrum subtracted from the 1.6 K spectrum (squares) with equivalent simulation (solid line). c) 3.2 Å spectra at 1.8 K (grey circles) and 15.0 K (black triangles). d) Intensity as a function of the neutron momentum transfer for excitation II. Solid lines are simulations based on the Hamiltonian and parameters given in the text, non‐liner background functions are represented as broken lines.

Further measurements performed on the IN5 spectrometer probed a broader dynamic range. Measurements with a 5.0 Å setting confirm the spectra from FOCUS with enhanced energy resolution, such that transition **I** is clearly resolved (Figure 4 a). A high‐resolution (8.0 Å) instrument setting enables the separation of transition **I** from the elastic line and at 6.0 K the equivalent excitation is also observed at negative neutron energy transfers. The temperature dependence of **I** and **II** clearly identifies the transitions as cold excitations. With a shorter neutron wavelength of 3.2 Å additional cold excitations labelled **III** and **IV** are accessed, with peak centres at 2.5 and 3.6 meV, respectively. From Figure 4 a it becomes evident that transition **II** exhibits a slight asymmetry, which is not evident for transition **I**. The neutron momentum transfer of **II** has a maximum at 1.2 Å^−1^ (Figure 4 d), consistent with the intermetallic distance between nearest neighbour metal ions within the Cr_8_Mn ring, relating to strong correlations between neighbouring spins within the cluster.

The magnetic data and the INS spectra in Figure 4 were simulated using a microscopic spin Hamiltonian [Eq. ((2))], in which ${\hat{𝐬}}_{1}$ to ${\hat{𝐬}}_{8}$ represent Cr sites with spin 3/2 and ${\hat{𝐬}}_{0}$ represents Mn with spin 5/2. *D*_Cr_ and *D*_Mn_ are the axial zero‐field parameters for the respective ions, *J*_CrCr_ is the isotropic exchange interaction between nearest neighbour Cr sites and *J*_CrMn_ is the isotropic exchange interaction between the Mn site and its neighbouring Cr sites.

(2) $$\hat{H}=J_{\mathrm{CrCr}}\sum_{i=1}^{7}{\hat{𝐬}}_{i}\cdot {\hat{𝐬}}_{i+1}+J_{\mathrm{CrMn}}\left({\hat{𝐬}}_{1}\cdot {\hat{𝐬}}_{0}+{\hat{𝐬}}_{8}\cdot {\hat{𝐬}}_{0}\right)+D_{\mathrm{Cr}}\sum_{i=1}^{8}\left({\hat{s}}_{z,i}^{2}-\frac{1}{3}s_{i}\left(s_{i}+1\right)\right)+D_{\mathrm{Mn}}\left({\hat{s}}_{z,0}^{2}-\frac{1}{3}s_{0}\left(s_{0}+1\right)\right)-g_{\mathrm{Cr}}\mu_{B}\sum_{i=1}^{8}𝐇\cdot {\hat{𝐬}}_{i}-g_{Mn}\mu_{B}𝐇\cdot {\hat{𝐬}}_{0}$$

The structural similarity of Cr_8_Mn with other Cr‐based rings provides a well‐defined starting point. The nearest neighbour Heisenberg exchange couplings, *J*_CrCr_ and *J*_CrMn_, are the dominant terms in the Hamiltonian; there is no justification for including longer range couplings. The magnetic data can be fitted with the parameters: *J*_CrCr_=1.32 meV, *J*_CrMn_=1.28 meV with *g*_Cr_=1.96 and *g*_Mn_=2.0. These parameters also allow us to simulate the main INS features. However, to obtain the precise position for the INS transition **I** and to correctly describe the splitting of transition **II**, an additional exchange parameter needs to be introduced. The simplest choice, which also keeps the overall *C*_2_ symmetry of the Hamiltonian, is to introduce the additional free parameter between Cr ions ${\hat{𝐬}}_{4}$ and ${\hat{𝐬}}_{5}$ . Slightly increasing the exchange between these two ions by *J*_4–5_/*J*_CrCr_=1.05 brings the simulated INS peak **I** into position with the measured results and reproduces the asymmetry of transition **II** well. Since the measured transitions are much broader than expected from the instrument resolution, the width of the peaks (assumed to be Gaussian) has been determined by fitting the experimental data.

**Figure 5:**
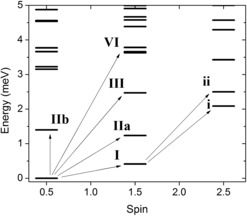
Calculated low‐lying isotropic exchange energy levels for Cr_8_Mn against spin number; arrows label the transitions identified by INS.

The calculated energies of the lowest total spin multiplets of Cr_8_Mn and the observed INS transitions are shown in Figure 5. As *J*_CrCr_ is similar to *J*_CrMn_, Cr_8_Mn is characterised by an *S*=1/2 ground doublet due to the competition between exchange interactions within the odd‐membered antiferromagnetic ring. We note that the additional *J*_4–5_ parameter splits the transitions labelled as **II a** and **II b**, reproducing the asymmetry in the measured peak.

The broadening of the measured INS peaks hinders the determination of the small anisotropy terms of the Hamiltonian. The single ion *D*_Cr_ and *D*_Mn_ values for the axial anisotropy obtained by INS measurements on similar compounds (e.g., Cr_8_Zn;^18^ *D*_Cr_=−28 μeV and Cr_7_Mn;^19^ *D*_Mn_=−3 μeV) were included within the Hamiltonian and are compatible with the simulation of INS results.

The effective ZFS $D_{S}=\sum_{i=0,8}\gamma_{i}D_{i}$ for the lowest spin multiplets in Cr_8_Mn and Cr_8_Ni rings compared to their non‐frustrated bipartite counterparts^19^ (Cr_7_Mn and Cr_7_Ni) reflects the non‐collinear internal spin structure. In the nine‐membered metal rings the projection coefficients,^20^ $\gamma_{i}$ , linking single ion terms with the ZFS of low‐energy‐spin manifolds, change sign around the ring in contrast to the bipartite case, in which sub‐lattices align parallel with each other, thus adding constructively. We have calculated *D_S_* for the lowest *S*>1/2 spin multiplet of some odd‐ and even‐numbered anti‐ferromagnetic rings using *D*_Cr_ and *D*_Mn_ derived from Cr_7_Mn.^19^ The axial anisotropy calculated for the first excited state of Cr_8_Mn (characterised by *S*=3/2) is *D*_*S*=3/2_=−0.00978 meV, and is much smaller than that for the related bipartite counterparts Cr_7_Ni and Cr_7_Mn in which *D*_*S*=3/2_=0.073855 meV and *D*_*S*=1_=−0.05962 meV, respectively. The projection of single ion anisotropies onto the low‐lying spin states was also found^21^ to be very small for Cr_8_Ni despite the large Ni single‐ion anisotropy term.

The energy level scheme classifies Cr_8_Mn as a Type II frustrated system.^15^ For Cr_8_Mn the bridging ligands are chemically equivalent around the ring, but the presence of the Mn^II^ *s*=5/2 spin breaks the rotational symmetry, thus leading to an isolated spin multiplet ground state. Since *J*_CrCr_ and *J*_MnCr_ are similar in magnitude the isolated spin ground state is characterised by *S*=1/2, thus classifying Cr_8_Mn as a Type II frustrated system. Other regimes of parameters, such as *J*_CrMn_ significantly less AF than *J*_CrCr_, would yield an *S*=5/2 ground state and consequently Type III frustration.

**Figure 6:**
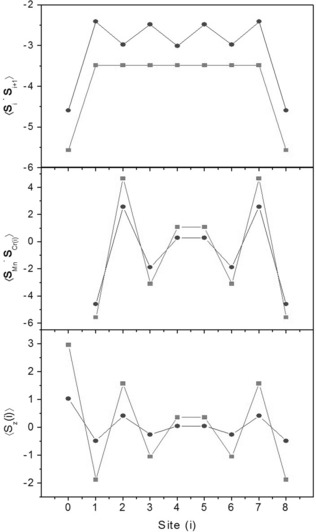
Spin pair correlations, for the quantum (circles) and classical (squares) spin case, within the *S*=1/2 spin ground state based on the exchange term in Hamiltonian Equation (1), in which Mn is at site 0 and equivalent to site 9. a) Nearest neighbour spin pair correlations around the Cr_8_Mn ring. b) Mn−Cr spin pair correlations. c) The local expectation values of *s*_z_ with an applied magnetic field of 0.1 T for each site *i*. The Mn ion tends to align parallel with the applied field causing the localisation of a node at the opposite side of the ring at sites *i*=4 and 5.

The microscopic Hamiltonian model describing the spin dynamics of Cr_8_Mn was used to investigate the nature of the ground state internal spin structure. Figure 6 a shows the calculated nearest neighbour spin‐pair correlations. These are stronger amongst the Cr1‐Mn‐Cr8 unit due to the larger spin moment of Mn^II^ (*s*=5/2) with respect to Cr^III^ (*s*=3/2). This creates a rigid Cr‐Mn‐Cr spin unit in which the spins are antiparallel and that couples to the remaining chain of Cr ions. Consequentially, non‐collinearity between the AF coupled spins is distributed around the remaining chain of Cr ions. Spin‐pair correlations between Mn and the eight Cr ions (Figure 6 b) further demonstrate this notion: the Mn−Cr1 and Mn−Cr8 correlations are large in magnitude, while $〈{\hat{𝐬}}_{\mathrm{Mn}}\cdot {\hat{𝐬}}_{\mathrm{Cr}4}〉$ and $〈{\hat{𝐬}}_{\mathrm{Mn}}\cdot {\hat{𝐬}}_{\mathrm{Cr}5}〉$ show expectation values close to zero, indicating a nearly perpendicular arrangement of the spins. Treating the spins as classical vectors (i.e., with length $\sqrt{s_{i}\left(s_{i}+1\right)}$ ) results in similar behaviour of the correlations with respect to the quantum spin model.

It is interesting to calculate the effect of an applied magnetic field on the ground state (i.e., in which *B* is significant but sufficiently less than the *S*=1/2 to *S*=3/2 crossing field). Figure 6 c shows how an external field breaks the spherical symmetry of the isotropic Hamiltonian: due the larger magnetic moment of Mn^II^ with respect to the Cr^III^ ions, the Mn ion tends to align parallel to the field thus producing a node on the opposite side of the ring.

**Figure 7:**
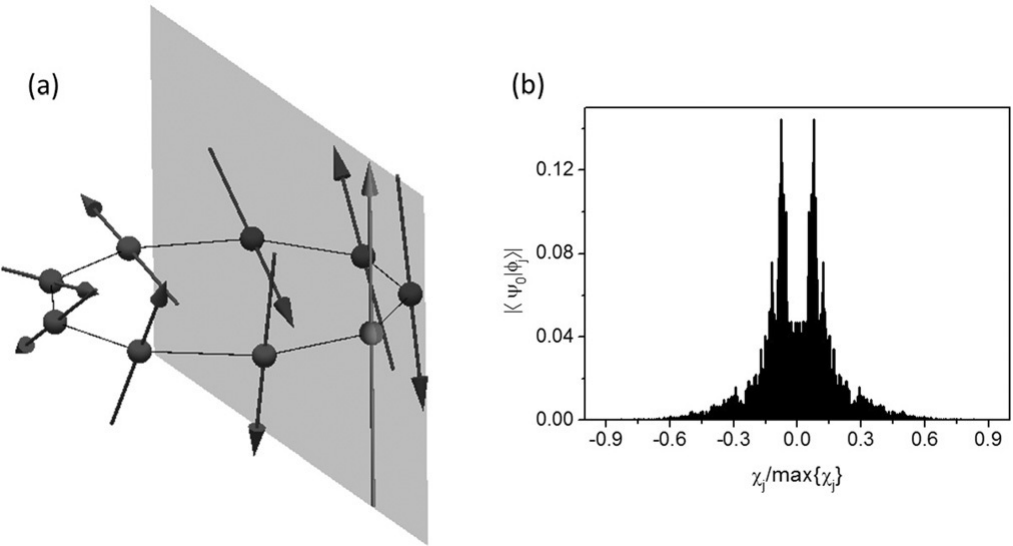
a) One of the configurations minimising the energy of the exchange part of the classical version of the Hamiltonian, Equation (2). The black and grey arrows represent Cr and Mn spins, respectively, with length $\sqrt{s_{i}\left(s_{i}+1\right)}$ . Due to the larger magnetic moment of the Mn ion (grey arrow) if compared to the Cr ions, the neighbouring Cr ions are locked in an almost collinear, antiparallel configuration. Moreover, a node is induced on the opposite side of the ring. All the spins belong to the same plane, as can be checked by computing the scalar chirality $\chi_{ijk}$ on each set of three spins *i*, *j* and *k*. b) Decomposition of the spin Hamiltonian ground state $|\psi_{0}〉$ onto the eigenstates $|\varphi_{j}〉$ of the chirality operator, $\hat{\chi }|\varphi_{j}〉=\chi_{j}|\varphi_{j}〉$ . The ground state results in an equal superposition of states with opposite chirality eigenvalues. Hence, in contrast with the classical situation (in which the chirality is zero, indicating a planar spin configuration), the ground state fluctuates between states with opposite chirality.

One of the classical configurations of minimum energy is shown in Figure 7 a, for the isotropic Heisenberg Hamiltonian. Due to the rotational invariance, this is only one of the infinite configurations minimising the classical energy. It is important to note that all the classical spin vectors lie on the same plane, that is, $\chi_{ijk}={\vec{s}}_{i}\cdot {\vec{s}}_{j}\times {\vec{s}}_{k}=0$ ∀i,j,k . This quantity, known as scalar chirality for the spins *ijk*, can be interpreted as a measure of the solid angle between the three spins. In order to investigate the planarity of the quantum spin state, we have decomposed the ground state spin wave‐function onto the eigenstates of the chirality operator $\hat{\chi }=\sum_{i}{\hat{𝐬}}_{i}\cdot {\hat{𝐬}}_{i+1}\times {\hat{𝐬}}_{i+2}$ (usual cyclic boundary conditions are applied). Results are shown in Figure 7 b: similar to the classical situation, the expectation value $〈\psi_{0}|\hat{\chi }|\psi_{0}〉$ vanishes in the ground‐state doublet. However, the quantum ground state results in an equal superposition of chirality eigenstates with opposite eigenvalues. Hence, the spin configuration in the quantum ground state fluctuates between non‐planar states corresponding to opposite eigenvalues of $\hat{\chi }$ .

### Muon spin relaxation

In a μSR experiment spin‐polarised positive muons are implanted into a sample.^22^ Muons have a mean lifetime of 2.2 μs. Each muon decays into a positron and two neutrinos. Due to the parity‐violating nature of the decay, the positron is preferentially emitted in the direction of the muon spin at the moment of decay and hence following the positron decay distribution as a function of time allows the determination of the time‐dependence of the muon spin polarisation. The measured quantity of the experiment is the asymmetry *A*(*t*)=(*N*_F_−*αN*_B_)/(*N*_F_+*αN*_B_), which is proportional to the average polarisation of the muon ensemble. Here *N*_F_ (*N*_B_) is the number of positrons recorded in detectors placed forward (backward) relative to the initial muon spin polarisation and *α* is a calibration constant. If the field distribution probed by the muon ensemble has non‐zero width, or if it fluctuates in time, then the muon spins will be depolarised. In large applied longitudinal fields, the muon spin will be decoupled from the electron and nuclear spins in the system, and the initial muon polarisation at implantation is preserved throughout the measurement. However, at certain values of the magnetic field, an avoided crossing of energy levels may occur, which leads to a loss of muon polarisation.^22^, ^23^

**Figure 8:**
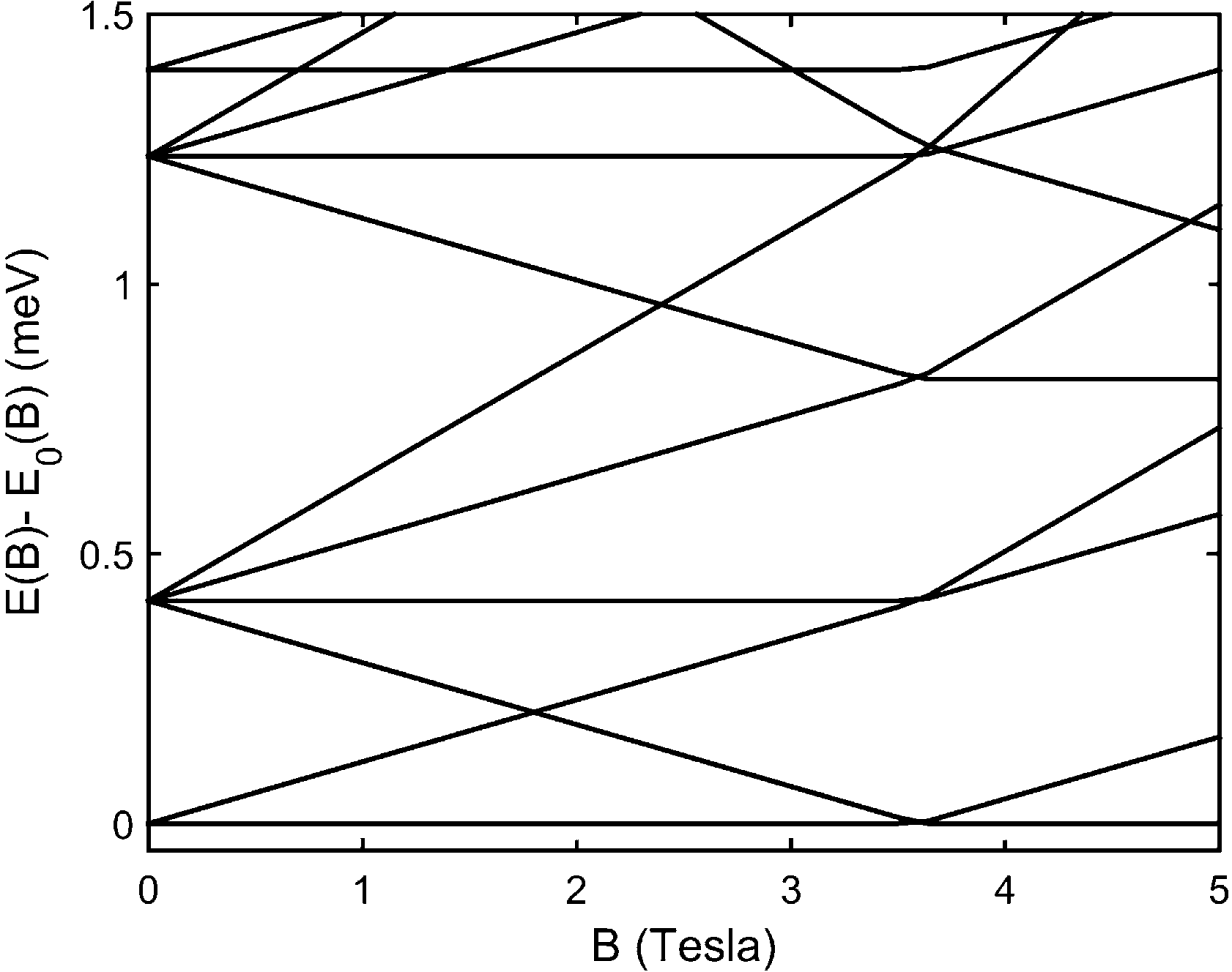
Cr_8_Mn Zeeman splitting of low lying energy levels with applied magnetic field. A change in spin ground state from |*S*=1/2, *Ms*=1/2〉 to |*S*=3/2, *Ms*=3/2〉 is observed 3.7 T (isotropic Hamiltonian).

Muon spin relaxation was used to study the Cr_8_Mn spin ground state as a function of applied magnetic field. Equation (2) predicts a change of ground state at below 4 T (Figure 8), therefore we investigated the spin dynamics at this region. Avoided crossings have been identified in Cr_7_Ni rings by using torque magnetometry, and single‐crystal inelastic neutron scattering has shown that the molecular spin at the avoided crossing oscillates coherently.^5^ In Cr_7_Ni the critical field to achieve a change of ground state is >10 T. The more modest field required for Cr_8_Mn allows us to perform μSR experiments at the crossing point.

Previously we reported the first observation of a *S*=0→*S*=1 ground‐state crossing with muons.^24^ Although it is possible that muons might only be sensitive to the “switching on” of a magnetic state in crossings involving the *S*=0→*S*=1 transition, it should be expected that, as the Cr_8_Mn system is driven through the *S*=1/2→*S*=3/2 level crossing, the dynamic magnetic field fluctuations that accompany the transition should cause rapid depolarisation of muon spins in paramagnetic stopping states (i.e., muon states that involve hyperfine coupling to the electronic spin) and that this should lead to a dip of the asymmetry versus applied magnetic field, allowing us to detect the crossing.

**Figure 9:**
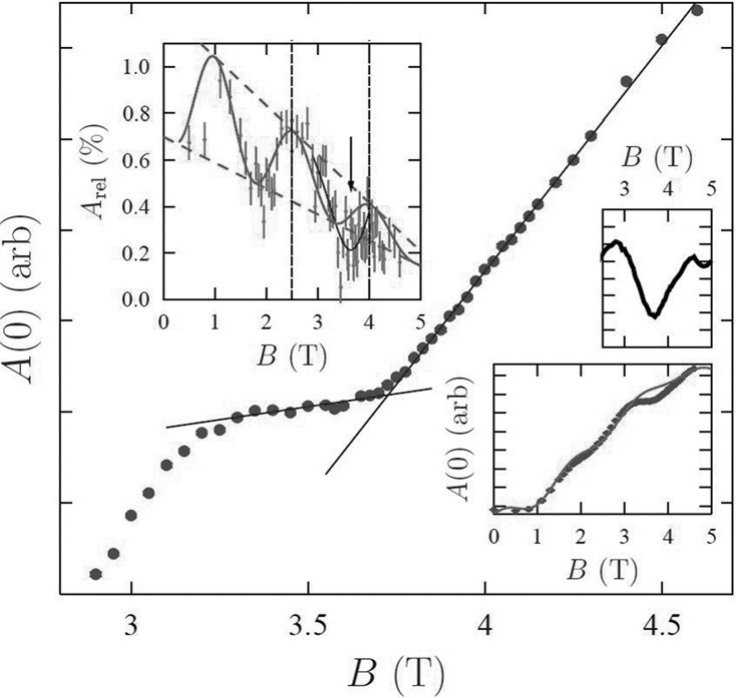
Main panel: Initial asymmetry A(*t*=0) as a function of applied magnetic field *B* in Cr_8_Mn, showing the influence of the level crossing around *B*=3.7 T. Right inset, bottom: A(*t*=0) shown across a wider field range shows a steady increase with increasing field and undulations caused by variations in the muon beam spot size (the background contribution is shown in grey); top: the resonance after background subtraction. Left inset: the amplitude of a small, slowly relaxing signal shows the effect of the beam spot variations around the focusing points at 2.5 T and 4 T with a minimum at the resonance (expected background contribution shown in grey). All data were measured at *T*=100 mK.

The initial asymmetry *A*(*t*=0) measured as a function of applied magnetic field *B* at *T*=100 mK is shown in Figure 9, in which we see that the dominant contribution is a slow, periodic undulation with applied field. This behaviour of *A*(*t*=0) may be accounted for by noting that the application of *B* deflects the decay positrons with respect to the detector array and changes the effective value of *α* in the definition of *A*(*t*) (see above), compared to its value determined in zero field, causing the general trend of an increase in *A*(*t*=0) with applied field (right lower inset, Figure 9).

In addition, the size of the incoming muon beam at the sample varies with *B* leading to broad undulations, with minima expected at the focusing fields of 1.0 T, 2.5 T and 4 T^22^, ^23^, ^25^ (as shown in left inset, Figure 9, the amplitude of this undulation is reduced at high fields). This contribution, with its expected minimum around 4 T makes unambiguously identifying the precise field range of a level crossing from the μSR difficult. However, around 3.5 T we find that *A*(*t*=0) levels off, before rising sharply from around 3.7 T. Although it is difficult to precisely account for the background (resulting from the effects of the magnetic field on the muon beam spot and positron trajectories), this feature suggests a level crossing in Cr_8_Mn. Indeed, the peak is larger than the expected variation in background in this field range. If we interpret the data in this way then, assuming linear variation of *A*(*t*=0) either side of the crossing, we estimate this occurs with a centre at *B*_cross_=3.73(5) T. Since we expect a minimum in the asymmetry as a function of the applied field, in correspondence of the level crossing,^22^ superimposing this minimum on the increase in *A*(*t*) caused by the field‐dependent change in *α* would then result in the behaviour we observe. Assuming this model, then a subtraction of an estimated background contribution (grey line, bottom‐right inset, Figure 9) leads to the resonance shown in the upper panel of the right inset, Figure 9, which has a full width half maximum of around 0.5 T. The precise width of a resonance derived in this way is highly dependent on the model used to fit the background, but is generally much larger than that expected for a pure level crossing involving states of different symmetries.

We also expect a minimum in relaxation rate at the crossing field, contributed by those muons in diamagnetic stopping states (i.e., muon states coupled to the electronic spin through a dipolar interaction). This reflects the increased magnetic fluctuations at the level crossing producing a motionally narrowed response at these muon stopping states and on resonance the apparent relaxation amplitude becomes reduced. Our results suggest such a contribution to the μSR signal comes from a very small relaxing component *A*_rel_, the amplitude of which is also strongly affected by the beam spot size effects, with maxima observed at the focusing fields (left inset, Figure 9). After subtracting the same periodically modulated background as assumed above, there is some evidence for the expected minimum at the crossing field, with a similar width to that seen in the paramagnetic contribution. We also note a change in relaxation rate of this component as we pass through the crossing region, which is also indicative of the crossing.

If the states at the level crossing become mixed due to symmetry lowering, quantum oscillations of the electronic spin may occur resulting in an avoided state crossing. In general, muon spin‐flipping can occur if there is significant amplitude in the spectral density of the electronic spin fluctuations, evaluated at the frequency corresponding to the muon Zeeman splitting in the local field. In this case of a transition around 4 T and a diamagnetic muon state, we require spectral density in the region of 1 μeV. We have shown previously that the muon is sensitive to the dynamics of the electronic spin in a molecular nanomagnet, but, crucially, that on the muon timescale it is the dephasing of these electronic spins by the disordered nuclear moments in the material that causes the muon‐spin relaxation.^26^ It is likely that the stochastic field caused by nuclear moments plays a role in any crossing process that we detect and the electronic spin fluctuations probed will be incoherent. Since the dephasing of the electronic spins probably represents the shortest timescale in the process, we would expect the transition probability of the electronic spins to be determined by the sum of instantaneous probabilities and therefore to be more complex than the Landau–Zener prediction. Regardless of the precise quantum oscillation mechanism, the width of a resonance in applied field provides an indication of the avoided crossing energy gap. The large resonance width of 0.5 T suggests the presence of an avoided crossing in the order of a few tens of μeV. Due to uncertainties in the field dependence of the background contributions to the measurement, a reliable estimate of the avoided crossing energy gap is not possible here. However a large resonance width, on the order suggested above, indicates the presence of an avoided crossing.

The Hamiltonian in Equation (2) with the parameters determined from INS and magnetisation results does not produce an avoided crossing. The inclusion of a Dzyaloshinski–Moriya interaction (DMI) term within the spin Hamiltonian can produce an avoided crossing in the order of several tens of μeV as suggested by μSR. For instance, a uniform DMI parallel to *Z* (normal to the plane of the Cr_8_Mn ring), with coupling constant of *J*/50, leads to an anti‐crossing of about 0.05 meV. For simplicity, we have included only the *Z* component of a DMI, which is also the most important in producing a level repulsion. We finally note that, while the introduction of a DMI within the microscopic model is the easiest way to induce a sizeable level repulsion, other mechanisms, such as a variance in the single‐ion anisotropy terms around the ring, would also create a small avoided crossing.

## Conclusions

The two Cr_8_Mn variants (**1 a** and **1 b**) differ in the way molecules are packed within the unit cell. The magnetic properties of the samples are found to be identical confirming that the magnetism of the crystalline samples is of molecular origin. A detailed spectroscopic investigation was pursued on **1 a**. INS measurements precisely characterise the low‐energy spin dynamics of Cr_8_Mn, showing an *S*=1/2 ground state. The results are accurately reproduced by a microscopic spin Hamiltonian with Heisenberg antiferromagnetic nearest neighbour exchange interactions, the magnitude of which is similar to other double‐carboxylate‐ and single‐fluoride‐bridged Cr^III^ rings. The heterometallic nature of the ring breaks its translational symmetry resulting in an isolated *S*=1/2 ground state. Calculations of the spin‐pair correlations within the ground state give the internal spin structure of Cr_8_Mn and it is found that the region around the Mn site has an anti‐ferromagnetic arrangement of spins, and spin non‐collinearity is distributed around the Cr ion segment of the ring. It is calculated that in an applied magnetic field the Mn^II^ ion moment partially aligns with the field vector creating a node localised at the opposite side of the ring. A classical calculation yields a planar configuration of minimum energy, while the quantum ground state dynamics is found to fluctuate between non‐planar states with opposite scalar chirality. μSR measurements performed as a function of the magnetic field suggest an *S*=1/2→*S*=3/2 ground state avoided crossing centred at 3.73(5) T. The crossing field is consistent with the model developed for the description of the INS results; however, a small DMI has to be included in the Hamiltonian to create an avoided crossing there. The INS simulations are insensitive to a small DMI term, whereas μSR spectroscopy in the region of the crossover field shows its potential as a probe of electronic transitions at level crossings in molecular nanomagnets.

Even‐membered antiferromagnetic rings have been proposed as candidates for quantum information processing^27^ and synthetically linking rings has been realised for the design of prototype qubits.^28^, ^29^ In such systems, magnetic fields are necessary to control entanglement and in general to implement quantum gates. Conversely, external electric fields can provide a more localised method to control spin states with opposite chirality, and such a methodology has been proposed and evaluated for the case of spin frustrated Cu_3_ triangles.^30^ The prediction of a fluctuating scalar chirality within the spin frustrated ground state of Cr_8_Mn highlights the necessity for further investigations of spin chirality within other odd‐membered odd‐electron molecular nanomagnets.

## Experimental Section

### Materials and general methods

Unless stated otherwise, all reagents and solvents were purchased from Sigma–Aldrich and used without further purification. Manganese(II) carbonate, Puratronic, 99.985 % (metals basis) was from Alfa Aesar. The Erlenmeyer Teflon FEP flasks were supplied by Fisher. Analytical data were obtained by the microanalysis laboratory at the University of Manchester—carbon, hydrogen, nitrogen analysis (CHN) by a Flash 2000 elemental analyser and metals analysis by Thermo iCap 6300 inductively coupled plasma optical emission spectroscopy (ICP‐OES). Electrospray ionisation mass spectrometry (ESI‐MS) were recorded on Micromass “QTOF Micro” quadrupole time of flight mass spectrometer.

**[H_2_N** ^***i***^ **(C_3_H_7_)_2_][Cr_8_MnF_9_(O_2_C*t*Bu)_18_] (1)**: Pivalic acid (30.0 g, 293 mmol), diisopropylamine (1.25 g, 12.4 mmol), and chromium(III) fluoride tetrahydrate (5.0 g, 28 mmol) were stirred for 4 h in an Erlenmeyer Teflon FEP flask in an oil bath at 160 °C. To this solution manganese(II) carbonate (0.75 g, 6.5 mmol) was added in small portions over a period of about 10 min and the reaction mixture was stirred for a further 66 h at 160 °C in a slow flow of N_2_. The flask was then allowed to cool to room temperature, acetone (50 mL) was added and the resulting mixture stirred for 2 h. The precipitate was collected by filtration, washed with acetone (ca. 300 mL) and dried in air. Then it was dissolved in pentane (100 mL) and filtered. The filtrate was diluted with toluene (50 mL) and the solution was concentrated by distillation to half of its initial volume, and allowed to cool to ambient temperature in a partially open flask. After concentration by slow evaporation during two weeks dark green hexagonal shaped crystals of **1** formed as a toluene solvate **1⋅**2 C_6_H_5_CH_3_ (**1 a**). The crystals were collected by filtration, washed with toluene (3×10 mL) and dried in air. Yield: 4.0 g (42 % based on Cr). Elemental analysis calcd (%) for C_110_H_194_Cr_8_F_9_MnNO_36_: Cr 15.13, Mn 2.00, C 48.07, H 7.11, N 0.51; found: Cr 15.11, Mn 2.02, C 47.99, H 7.31, N 0.52. ESI‐MS (sample dissolved in THF, run in MeOH): *m*/*z* (%): 2461.6 [*M*−(O_2_C*t*Bu)]^+^, 2563.3 (100) [*M*+H]^+^, 2585.2 [*M*+Na]^+^, 2665.0 [*M*+(C_3_H_7_)_2_NH_2_]^+^.

**[H_2_N** ^***i***^ **(C_3_H_7_)_2_][Cr_8_MnF_9_(O_2_C*t*Bu)_18_]⋅H_2_O (1 b)**: The packing and relative orientation of Cr_8_Mn molecules in the crystallographic unit cell of **1 a** can be altered to give **1 b**. Several large crystals of **1 a** were dissolved in a minimum amount of ethyl acetate at room temperature and the solution was diluted drop by drop with MeCN until the solution become cloudy. The solution was gently heated (ca. 50 °C) and a little ethyl acetate was added to re‐dissolve the microcrystalline precipitate. The clear solution was allowed to cool and was left in a partially open flask at room temperature. Small dark green crystals started to form over 2 days and were collected (including X‐ray quality crystals of 1 H_2_O) after two weeks. Elemental analysis (%) calcd for C_96_H_180_Cr_8_F_9_MnNO_37_: Cr 16.11, Mn 2.13, C 44.65, H 7.03, N 0.54; found: Cr 15.95, Mn 2.14, C 44.84, H 7.04, N 0.83. ESI‐MS (**1 b** dissolved in THF, run in MeOH) was the same as for **1 a**.

**Crystallographic data collection**: For compound **1 a** crystallographic data was recorded on a X8 prospector Bruker SMART CCD diffractometer with Cu_Kα_ radiation (*λ*=1.54184 Å) at a temperature of 100 K, equipped with an Oxford Cryosystems Cobra nitrogen flow gas system. X‐ray data for **1 b** were collected at Diamond Light Source beamline I19 (*λ*=0.6889 Å),^31^ at a temperature of 30 K. Data were measured using CrystalClear‐SM Expert 2.0 r5 suite of programs.

**Crystal structure determinations and refinements**: X‐ray data were processed and reduced using the CrysAlisPro suite of programs. An absorption correction was performed using empirical methods based upon symmetry‐equivalent reflections combined with measurements at different azimuthal angles.,^32^, ^33^ The crystal structure was solved and refined against all *F* ^2^ values using the SHELXTL suite of programs.^34^ For compound **1 a**, only the metal sites and fluoride atoms could be found due to the extremely high disorder found. No attempt was made to refine the structure to convergence. Crystal data for **1 a**: formula C_110_H_194_Cr_8_F_9_MnNO_36_, hexagonal, space group *P*6_3_/*m*, *T*=100(2) K, *a*=*b*=19.552(2), *c*=24.489(3) Å.

The crystallographic data and experimental details of the structural refinement for the X‐ray crystal structure of **1 b** are given in the Supporting Information. CCDC 1402276 contains the supplementary crystallographic data for this paper. These data are provided free of charge by The Cambridge Crystallographic Data Centre.

**Magnetic measurements**: The magnetic properties of polycrystalline samples of **1 a** and **1 b** were measured with a Quantum Design MPMS‐XL7 SQUID. The samples were ground, placed in a gel capsule and fixed with a small amount of eicosane to avoid movement during the measurement. The data were corrected for the diamagnetism from the gel capsule and the eicosane with the diamagnetic contribution from the complexes calculated from Pascal constants.

**Inelastic neutron scattering (INS) studies**: Time‐of‐flight INS measurements on non‐deuterated polycrystalline samples of **1 a** were performed at the IN5 spectrometer at Institute Laue‐Langevin,^35^ Grenoble (France) and the FOCUS spectrometer at the Swiss spallation neutron source SINQ, Paul Scherrer Institute^36^ (Switzerland). The sample of **1 a** was sealed inside a hollow aluminium cylinder for measurement. Measurements were performed at various temperatures within a range from 1.5–18 K. IN5 data were measured with several chopper settings, for which different speeds and ratios were used to select the optimum resolution, energy‐ and momentum‐transfer ranges.

INS energy spectra were obtained by integration of scattering intensity over all detector angles (−12 to 135° and 10 to 130° in horizontal primary scattering plane for IN5 and FOCUS, respectively). Detector efficiencies were normalised to a standard vanadium measurement.

### Muon spin relaxation (μSR) spectroscopy

Muon spin relaxation measurements were performed on **1 b** using the HiFi spectrometer at the ISIS facility, Rutherford Appleton Laboratory (UK).25a Two unaligned crystals of Cr_8_Mn were mounted on the cold‐finger of a dilution refrigerator. All measurements were performed at 100 mK with a longitudinal magnetic field applied parallel to the initial muon spin polarisation.

## Supporting information

As a service to our authors and readers, this journal provides supporting information supplied by the authors. Such materials are peer reviewed and may be re‐organized for online delivery, but are not copy‐edited or typeset. Technical support issues arising from supporting information (other than missing files) should be addressed to the authors.

Supplementary
