# Supplementary material for: Studies of a Large Odd‐Numbered Odd‐Electron Metal Ring: Inelastic Neutron Scattering and Muon Spin Relaxation Spectroscopy of Cr8Mn
Source: Chemistry. 2016 Jan 8;22(5):1779–88. doi: 10.1002/chem.201503431 (PMC4744977; doi:10.1002/chem.201503431)
Supplement: Supplementary file 1 — Supplementary [file CHEM-22-1779-s001.pdf]

CHEMISTRY
A European Journal

Supporting Information

Studies of a Large Odd-Numbered Odd-Electron Metal Ring: Inelastic Neutron Scattering and Muon Spin Relaxation Spectroscopy of Cr_8Mn

Michael L. Baker,*[a, b] Tom Lancaster,[c] Alessandro Chiesa,[d] Giuseppe Amoretti,[d] Peter J. Baker,[e] Claire Barker,[a] Stephen J. Blundell,*[f] Stefano Carretta,[d] David Collison,[a] Hans U. Güdel,[g] Tatiana Guidi,[e] Eric J. L. McInnes,[a] Johannes S. Möller,[f] Hannu Mutka,[b] Jacques Ollivier,[b] Francis L. Pratt,[e] Paolo Santini,[d] Floriana Tuna,[a] Philip L. W. Tregenna-Piggott†,[g] Iñigo J. Vitorica-Yrezabal,[a] Grigore A. Timco,[a] and Richard E. P. Winpenny*[a]

chem_201503431_sm_miscellaneous_information.pdf

Crystallographic Study of 1b

For compound **1b** with formula $C_{96}H_{180}Cr_8F_9MnNO_{37}$ all atoms except the oxygen corresponding to the water molecule and hydrogens were refined anisotropically. Hydrogen atoms were placed in calculated positions refined using idealized geometries (riding model) and assigned fixed isotropic displacement parameters. The C-C and C-O distances in the pivalate ligands were restrained using DFIX and SADI command. The atomic displacement parameters (adp) of the ligands have been restrained using RIGU, EADP and SIMU commands.

Table S1. Crystallographic information for **1b**

	**1b**
Crystal colour	Green
Crystal size (mm)	0.25 × 0.25 × 0.1
Crystal system	Monoclinic
Space group, *Z*	$P2_1/n$, *4*
a (Å)	19.3268(5)
b (Å)	22.2588(4)
c (Å)	31.1087(9)
β (°)	90.743(2)
V (Å^3)	13381.6(6)
Density (Mg.m^{-3})	1.282
Wavelength (Å)	0.6889
Temperature (K)	30
μ(Mo-Kα) (mm^{-1})	0.735
2Θ range (°)	3.728 to 49.038
Reflns collected	83088
Independent reflns (R_{int})	24219
L.S. parameters, *p*	1771
No. of restraints, *r*	1864
$R1$ $(F)^a$ $I > 2.0\sigma(I)$	0.0805
$wR2(F^2)$,[a] all data	0.2283
$S(F^2)$,[a] all data	1.028

[a] $R1(F) = \Sigma(|F_o| - |F_c|)/\Sigma|F_o|$; [b] $wR^2(F^2) = [\Sigma w(F_o^2 - F_c^2)^2/\Sigma wF_o^4]^{½}$; [c] $S(F^2) = [\Sigma w(F_o^2 - F_c^2)^2/(n + r - p)]^{½}$
